# Optimize data-driven multi-agent simulation for COVID-19 transmission

**DOI:** 10.1186/s12859-022-04799-4

**Published:** 2022-07-01

**Authors:** Chao Jin, Hao Zhang, Ling Yin, Yong Zhang, Sheng-zhong Feng

**Affiliations:** 1 National Supercomputing Center in Shenzhen, Shenzhen, 518055 Guangdong People’s Republic of China; 2 Shenzhen Institute of Advanced Technology, Chinese Academy of Sciences, Shenzhen, 518055 Guangdong People’s Republic of China; 3 University of Chinese Academy of Sciences, Beijing, 100049 People’s Republic of China

**Keywords:** Multi-agent simulation, Case-focused method, Hash table

## Abstract

**Background:**

Multi-Agent Simulation is an essential technique for exploring complex systems. In research of contagious diseases, it is widely exploited to analyze their spread mechanisms, especially for preventing COVID-19. Nowadays, transmission dynamics and interventions of COVID-19 have been elaborately established by this method, but its computation performance is seldomly concerned. As it usually suffers from inadequate CPU utilization and poor data locality, optimizing the performance is challenging and important for real-time analyzing its spreading.

**Results:**

This paper explores approaches to optimize multi-agent simulation for COVID-19 disease. The focus of this work is on the algorithm and data structure designs for improving performance, as well as its parallelization strategies. We propose two successive methods to optimize the computation. We construct a case-focused iteration algorithm to improve data locality, and propose a fast data-mapping scheme called hierarchical hash table to accelerate hash operations. As a result, The case-focused method degrades $\sim 90 \%$ cache references and achieves $\times 4.3$ speedup. Hierarchical hash table can further boost computation speed by 47%. And parallel implementation with 20 threads on CPU achieves $\times 80$ speedup consequently.

**Conclusions:**

In this work, we propose optimizations for multi-agent simulation of COVID-19 transmission from aspects of algorithm and data structure. Benefit from improvement of locality and multi-thread implementation, our methods can significantly accelerate the simulation computation. It is promising in supporting real-time prevention of COVID-19 and other infectious diseases in the future.

## Background

Multi-agent simulation (MAS) is an essential technique for exploring system phenomena in which the overall behaviour is determined by the constituent autonomous entities [1]. It provides an effective tool for modeling systems with complex organization and non-linear interactions. As agents can be applied with different disciplines, it is widely used in studying social, economic, organization and epidemiology sciences [2–4]. Domínguez et al. [5] propose multi-agent modeling for complex supply chains to overcome limitations from classical methods. McArthur et al. [6] investigate MAS technology in power industry with complex scalable power networks. Moreover, it is also evaluated as efficient in smart city, such as controlling city congestion, pollution and delivery time [7]. Many other applications can be found in [8].

A significant application of MAS is studying infectious diseases. Classical methods use mathematical models to emulate the transmission of infectious diseases. Some classical examples include Susceptible-Infect-Susceptible (SIS) epidemic model [9], Susceptible-Infect-Recovered (SIR) epidemic model [10], etc. However, these simple models are not afforded to analyze complex and find-grained systems. MAS integrating evolution and phylogeny helps to understand emerging infectious diseases in complex systems. Dion et al. [11] leverage it to study the landscape epidemiology of the foot-and-mouth disease in South Africa. Yergen et al. [12] propose IDESS for rapidly constructing MAS models of Avian Flu (H5N1) virus spreading.

Outbreaks of COVID-19 raise concerns about effectively preventing spread of infectious diseases, and MAS is widely adopted for studying this issue. Its fine-grained spreading dynamics is established through many efforts. Castro et al. [13] analyze the spread processes of COVID-19 epidemics in open regions by considering effects from different environments. Vyklyuk et al. [14] propose modeling its spread in large regions by simulating a set of autonomous multi-agent systems. Nanna et al. [15] extend MAS to dynamically verify influences on diseases spread from government strategies. For COVID-19 preventions, both non-pharmaceutical interventions (NPI) and pharmaceutical interventions (PI) have been elaborately invested [16] with this paradigm. Yin et al. [17] proposed a data-driven NPI MAS model to suppress the diseases in Shenzhen and evaluate strategies including contact tracing, mask wearing and prompt testing. Zhou et al. [18] examine the spatial heterogeneity of the disease transmission and optimize vaccine distribution strategies considering spatial prioritization.

Although many researches focus on exploring underlying dynamics through MAS modeling, fewer concern the computation performance [19–21]. However, computation performance is a significant aspect for large-scale simulations where obstructions from heavy calculation, intensive memory access and communication are inevitable. In general, approaches to improve MAS performance include parallel computing [22–24] and distributed computing [25–27] on both CPU and GPU platforms. Well known of parallel and distributed platforms contain Mason [28], Gama [29] and Simphony [30]. Meanwhile, FPGA is remarked with fine-grained parallelism and flexible memory architecture. Some studies also focus on the acceleration on FPGA platforms [31, 32]. As communication patterns among agents are continuously changing in distributed systems, some works seek for effective agent allocation strategies to improve the performance [33, 34]. On the other hand, massive-scale simulations in serial algorithm often suffer from poor data locality, but seldom researches about this issue is established. Willem et al. [35] introduce a sorting phase of population and optimize data structure to improve system performance.

A general MAS simulation [36] for infectious diseases contains two steps: (a) update each person’s health state according to the epidemic model; (b) compute the disease transmissions over the contact network. Accessing agent states is irregular and random, which is the major factor limiting the computational efficiency. In this work, we study optimizing MAS for COVID-19 transmission in Shenzhen. Two methods are explored and evaluated including algorithm and data structure optimization. In order to improve data locality, we reconstruct the loop order of MAS algorithm, and propose a hierarchical Hash table structure leveraging cache and hardware properties. Our results show prominent improvement in the system performance and indicate wide applicability to interventions for infectious diseases.

Specifically, we make the following contributions:

1. We reorder the inner structure of MAS method to improve the data locality, where iterations of infectious agents take priority rather than the time evolution. And formulate a simple convolutional scheme to eliminate its systematic errors. (Section Methods–Case-focused simulation)
2. We propose a hierarchical hash table to support irregular and randomly accessing agent states with high efficiency. It leverages cache characteristics to organize data in a compact manner and reduce its sparsity in memory space, and adopts a key-value separating structure for flexible operations. In addition, single instruction, multiple data (SIMD) instructions are applied to boost the handling speed of hash collisions. (Section Methods– Hierarchical Hash Table)

## Methods

### Epidemic dynamics

A stochastic, discrete-time susceptible-latent-infectious-removed (SLIR) model is implemented where the transmission of COVID-19 is triggered by contacts between agents in households, workplaces, schools and other buildings [17]. Once a susceptible individual has a contact with an infectious agent, the probability of infection *p* via this contact is calculated as follows:

1 $$\begin{aligned} p = pTrans \times I_c \times r \end{aligned}$$

where *pTrans* denotes the transmission probability per contact and is estimated as 0.165 by calibrating the modeled basic reproductive number $R_0$ to the observed value of 2.4 [37–41]. $I_c$ is the intensity of daily contact at different contact settings derived from a contact survey [42]. *r* differentiates the infectivity of infectious agents with and without symptoms, i.e. the infectivity of asymptomatic agents is as 0.12 of their counterparts [17]. In addition, this simulation assumes that all infected agents would not be re-infected.

**Fig. 1:**
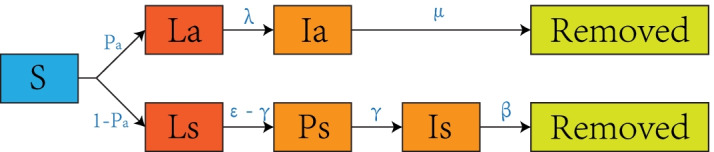
A compartmental SLIR model for COVID-19

The infectious dynamics of SLIR model is demonstrated in Fig. 1. Once a susceptible individual (S) is infected, a probability of 25% is assumed to turn into latent status [43–45]. The latent period (La) is set to 4.6 days ($\lambda$) for asymptomatic agents to become infectious [46–48]. These agents remain infectious for 9.5 days ($\mu$) until being removed from the model after recovery [49]. Symptomatic agents are assigned an incubation period ($\epsilon$) with a mean of 5.2 days to manifest symptoms (Is), including latent status (Ls) [48]. Their infectivity starts from 2 days ($\gamma$) before symptom onset (Ps) [47]. After the onset of symptoms, agents remain infective until they get recovered. Parameters in this SLIR model are confident based on the assessment of local Center for Disease Control (CDC) collaborators, who have first-hand COVID-19 clinical data.

### Case-focused simulation

In general, MAS algorithm for infectious diseases makes efforts to mimic its natural transmission process among population, where diseases spread as time evolves. Hence, traditional MAS algorithm is evolution-focused, where the evolution is represented as calculation by time step, and is shown in Algorithm 1. It contains 3-order loops. The first loop ($k \in MaxIter$) is the time-evolving loop, where *MaxIter* is simulated max time step. The second loop is made up by three subprocesses (two of $a_i(k) \in A(k)$, and one of $b_j(k) \in B(k)$), including removing recovered agents, building up contacting networks, implementing diseases spreading calculation. *A*, *B* organized as hash table format denote infected agents and contacting network respectively. *Regions* represents general venues population gather together including both private and public areas. The innerest loops ($g_i \in Regions(a_i)$ and $l_j \in b_j(k)$) scan over infected agents’ appearance places daily to find contacting population and contacted candidates to decide increasingly infected agents at current time step *k*. These agent information are dynamically changed with either shrinking or expanding behaviors in hash tables *A* and *B* during the iteration.
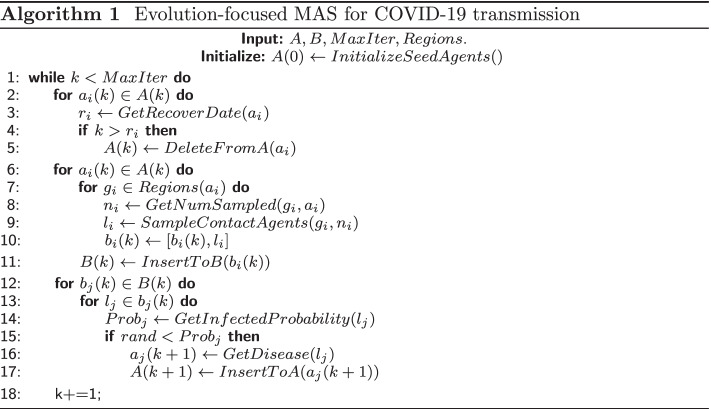

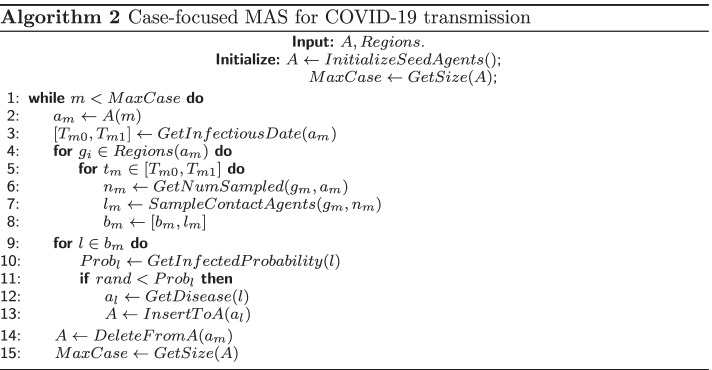


As indicated by middle-order loops in this algorithm, all the elements from the constructed hash tables *A* and *B* require accessing and utilization once for each time iteration. As the amount of population is large, such a scanning mechanism wastes data fetching from memories. It will degrade system performance severely owing to this poor locality as data arrangement heavily exceeds cache capacities. For simulation in a megacity with ones of millions population, this issue is inevitable in performing real-time simulation and quickly response to intervention strategies. Key point is to manage effective reutilization of data as fetching from hash tables to maintain the locality.

In order to improve data locality, we propose a case-focused method, where the algorithm loops are reorganized. As shown in Algorithm 2, the table scanning loop is elevated to the outer order, while time-evolution loop is demoted. *MaxCase* is size of *A*, and changes automatically as *A* varies during simulation. At a primitive transmission stage of pandemic diseases, rapid spreading among population increases *MaxCase* prominently. It will get decreased and vanish when diseases are suppressed and immunity gets common. In the reconstructed loops, infected agent $a_m$ gets accessed and transferred to cache once for each iteration. The time evolution is implemented in the inner loop $[T_{m0}, T_{m1}]$, thus $a_m$ can be reutilized $T_{m1} - T_{m0}$ times. Meanwhile, as infection period of $a_m$ finishes at the end of each agent *m*, *m* gets recovered naturally and we need not to query for recovered agents appearing at evolution-focused method. In addition, contacting candidates $b_m$ are sampled independently for each infected agent $a_m$, and construction of *B* is independent. As bulky information of agents will be transferred into higher-level cache and frequently fetching from main memory is suppressed, it is expected that case-focused method will improve system performance significantly.

It should be mentioned that these two algorithms have similar computation complexity possessing 3-order loops. An interesting difference between them is causality. The evolution-focused method follows a natural process, while case-focused method reduces priority of time evolution to maintain data locality. It will affect produced distribution of infected-agent evolution. For widely spreading diseases with large overlapping susceptible population from infectious agents, the probability of a candidate $b_i$ gets infected is boosted by both of the amount of its contacting infectious agents and their infectious periods. The calculation order of the two factors make different effects, where the inner loop of factor takes priorities. For each time step *k* in evolution-focused MAS, number of contacting agents take the priority to determine candidate’s healthy status. However, in case-focused MAS, each candidate agent *m* is domained by infectious agent’s period $[T_{m0}, T_{m1}]$. Both of them share a same infectious probability, but candidate $b_m$ is more likely to be infected within a relatively delayed time with respect to evolution-focused method.

We formulate this difference as the systematic error from case-focused method. It can be demonstrated and modified by a simply assumption. Assuming the delayed infected time $\delta$ follows a uniform distribution, it can be directly derived from statistic theory that the average delayed time $\bar{\delta }$ obeys a gaussian distribution $N(\mu , \sigma ^2)$. $\mu$ represents the mean of the distribution, and $\sigma ^2$ is its variance, which can be regarded as super parameters tuned for real simulation. Conversion between two algorithms can be implemented by convoluting former method with a delayed-gaussian function. In other words, case-focused MAS will generate delayed and vague results with respect to traditional method.

### Hierarchical hash table

**Fig. 2:**
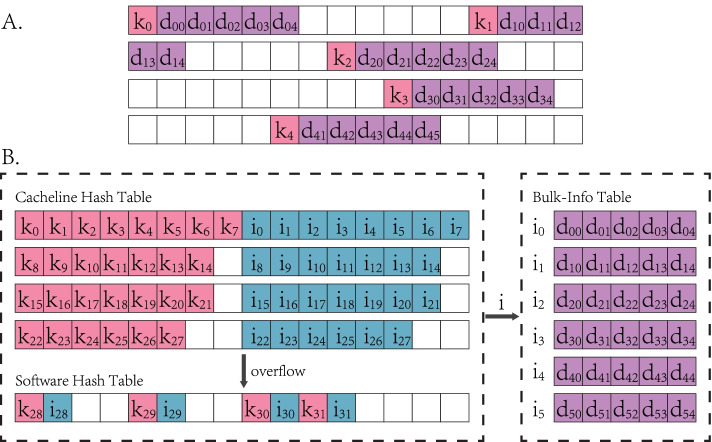
**A**: Traditional (software) hash table format. **B**: Overview of hierachical hash table format

As indicated in algorithms 1 and 2, hash table is an important participant in the simulation, and its performance affects system significantly. In general, hash tables consume the majority of cycles on many key applications such as databases [50], networking [51] and genomics [52]. But they suffer from inefficiencies in current systems owing to poor core utilization and poor spatial locality [53]. Hash tables spread key-value pairs uniformly and sparsely across allocated memory to reduce mapping conflicts. In the MAS simulation, each agent’s index and its bulky information are formed together into the key-value pair filled in the table as shown in Fig. 2A. Due to sampling for agents is a random process during the simulation, same-line neighbors of frequently accessed agents may be rarely accessed. This leads to a significant waste of cache capacity.

There are many researches focusing on reducing hash table overheads. Data-level parallelism of inter keys is introduced in prior works to optimize the throughput [50, 54]. Near-memory [55] and near-storage [56, 57] acceleration bypass the cache hierarchy entirely to avoid spatial locality problems. Exploiting hierarchical memory layout and characteristics of caches [53] is found improving spatial locality prominently.

In the MAS computation, the key-value pair of agent information is made up by agent index (key, *k*) and bulky data (value, *d*) derived from simulation, such as infected and recover date. These pairs stored by traditional (software) hash tables are allocated sparsely across memory as demonstrated in Fig. 2A. Lookup and update operators of hash table have to access bulk memory in units $k+d$, which generate larger memory occupancy and heavier accessing burden. Hence, cross-line and misses of caches are frequently confronted. It is essential to allocate hash table elements across memory into a dense alignment for better spatial locality.

We reorganize hash table as a hierarchical structure and leverage caches to optimize spatial locality. The agent table constructed during the simulation is organized as three-level hierarchical tables in Fig. 2B. This hierarchical hash table (HHT) is composed of three parts: Cacheline Hash Table (CHT), Software Hash Table (SHT) and Bulk-Info Table (BIT). Agent indices and bulky data are stored separately, while extra projection index *i* is introduced to connect them. CHT and SHT store (*k*, *i*) pairs, in which *i* indicates address offset to locate *d*. BIT is a compact array where agents’ information data *d*s are aligned contiguously and accessed by *i* transferred from former hash tables. Although projection indices possess extra memory, compact alignment of agent information data saves prominent memory occupancy. In addition, MAS computation queries the agent’s existence frequently leaving its information alone, such a key-value separating architecture is expected to save memory occupancies and boosts key-only lookup speed.

CHT leverages the characteristics of cache to accelerate hash operations. Data is transferred in fixed block size between cache and main memory, namely cache line. In nowaday processors, a typical cache line size is 64-bytes. We construct cache line as the basic CHT element, where hash values derived from keys are located to address of cache line. In a line, data-level parallelism is implemented, where first 32 bytes make up 8-key group and the rest is corresponding index group. As elements within a cache line share the same hash value, collision occurs when lookup CHT. We use SIMD instructions to handle this issue and accelerate lookup and update in the line. SHT is used as a victim table, which holds pairs overflowing from CHT. It has a traditional structure and occupies subtle space with respect to CHT to maintain CHT priority.

Major interfaces of HHT contain three hash-table operations: find, insert and delete. Find operator decomposes key-value querying requestion into two parts. CHT has the priority to search for the key. If CHT’s lookup is not resolved, i.e., the key is not found and the line is full, it continues searching across SHT. Key searching operation returns corresponding projection address *i* of *k*. If *i* is meaningful and bulky data is required, specific values are extracted as located at i in BIT. For implementing insert and delete operations, two extra labels are introduced representing their states. Insert operator fills content at empty seat of the line in CHT, and turns to SHT if a cache-line overflow occurs. Storage of the bulky value makes continuously increments at the end of BIT. Moreover, delete operation is achieved by replacing target content with the delete label. As a result, CHT and BIT make dense alignment for key-value storage and can reduce overhead for accessing cache.

## Results and discussion

The megacity Shenzhen with a population larger than ten million is adopted as a study case. We implement the optimization methods on spatial explicit MAS system without NPIs proposed by Yin [17]. In the system, 11.2 million agents with demographic characteristics are synthesized in assistance of cross-referencing census data and house-hold travel survey [58]. Agents’ hourly movements are formulated from mobile phone trajectory records or house-hold travel survey. Each individual is anchored onto different types of buildings for a daily trajactery, including living, working, studying and performing other activities. The synthesized agents are modeled to contact with each other when staying at the same location within one hour.

We perform the simulation on an Intel Core i9-10850K CPU platform with 10 physical cores and 20 threads. It is configured with 64 GB main memory, and cooperates with 32 KB L1 DCache, 256 KB L2 Cache and 20 MB L3 Cache. As case-focused MAS is convenient to be implemented in data-parallel manner, we perform multi-thread computation in assistant of C++11’s **thread**, **mutex** and **atomic** libraries. **thread** is used to create and detach threads for agent-parallel processing. **mutex** and **atomic** help to avoid changing memory values simultaneously among threads.

**Fig. 3:**
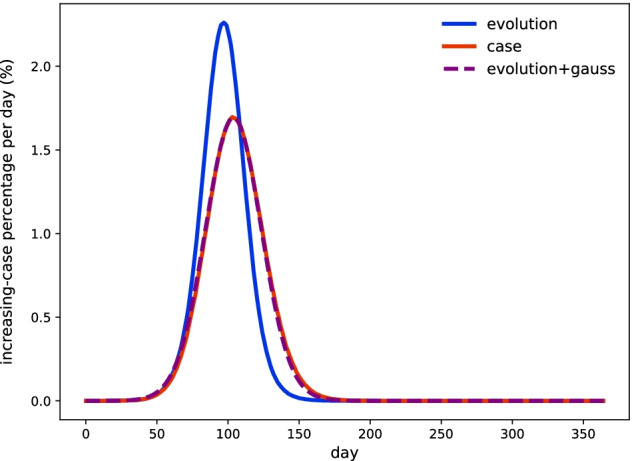
Evolutions of increasing cases per day among different methods. Blue solid line is calculated by evolution-focused MAS. Read solid line is calculated by case-focused MAS. Purple dashed line is the result from convoluting evolution-focused MAS outputs with delayed-gaussian function $N(6, 13^2)$

First, performances between Algorithm 1 and Algorithm 2 are present on single-thread mode. As shown in Table 1, both of these methods generate almost the same amount of total infected agents. But their time evolution diverges as demonstrated in Fig. 3. Convolution with gaussian function is implemented to eliminate divergences, and parameters are fitted with ROOT toolkit [59] as $N(6, 13^2)$. It results that average delayed time is 6 days with 13-day deviation. In the statistical perspective, 68% of cases would be observed with a delay of $6 \pm 13$ days as calculated by the case-focused MAS, and a typical confidence of 95% derives this range with $6 \pm 26$ days.

**Fig. 4:**
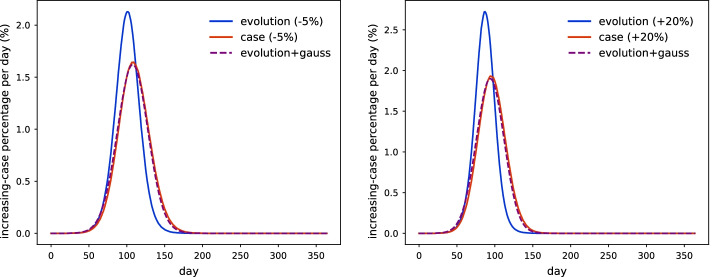
Evolutions of increasing cases per day among different methods with variances of infectious probability as -5% (Left) and +20% (Right). Blue solid lines are calculated by evolution-focused MAS. Read solid lines are calculated by case-focused MAS. Purple dashed lines are the result from convoluting evolution-focused MAS outputs with delayed-gaussian function $N(6, 13^2)$

We perform error analysis about parameters applied in the MAS. First, both values of infectious probability and period are assumed with 20% uncertainties. Simulation results indicate that they have similar effects on the amount of total infected agents, which generate variations of the amount within [−7%, 4%] respectively. Second, the stability of the gaussian kernel is evaluated under uncertainties of the infectious probability. We find parameters of the kernel can remain almost unchanged within its deviation [−5%, 20%] considering statistical uncertainties, and results are shown in Fig. 4. This systematic error of Algorithm 2 originates from overlapping contact networks of susceptible individuals under different iteration orders. It will have little effects as the infected agents possess a minor amount of the population. We produce this scenario by assuming in-time quarantined of infectious population to heavily suppress the diseases. Results demonstrate that both Algorithm 1 and Algorithm 2 are equivalent to each other under statistical uncertainties.

As mentioned above, case-focused iteration has potential to significantly improve system performance by maintaining data locality. We use **perf** provided by Linux to evaluate cache accesses during simulation. As shown in Table 1, results indicate that almost 94% cache references are saved and cache misses are reduced by 90%. This optimized algorithm accelerates simulation prominently and achieves $\times 4.4$ speedup. As a result, it maintains the data locality well and improves calculation speed significantly, but sacrificing part of precision.

**Fig. 5:**
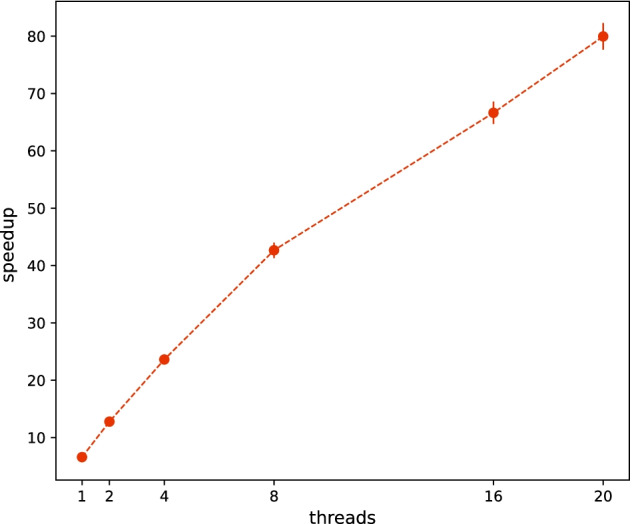
Multi-thread speedup of HHT implementation on the case-focused MAS comparing to benchmark (unordered_map implementation on the evolution-focused MAS)

Next, proposed hash table HHT is implemented for further optimizing system performance under the case-focused method. As SHT is provided by system and holds victims overflowing from CHT, frequently accessing SHT may affect HHT’s querying performance. We set its member occupancies is 1% to CHT capacity to minimize the influence. Algorithm 1 with libstdc++’s C++11 unordered_map under single-thread operation is adopted as benchmark. The single-thread computation is shown in the first ticks along thread axis of Fig. 5. HHT achieves $\times 6.4$ speedup which leverages both algorithm and hash table optimization. Comparing the result with unordered_map implemented under the same MAS algorithm, HHT is still faster by 47%.

Traditional hash table is a thread-unsafe data mapping paradigm. It requires multiple memory duplication and migration during the table expands or shrinks. As hash **insert** and **find** encounter from different threads, while the **insert** causes rehashing and expands the table’s capacity by factor 2, another thread may lose the actual access to target and a program fault occurs. We use atomic operator on HHT to maintain a thread-safe manner across multi-thread operations. As demonstrated by Fig. 5, this parallel implementation prominently boosts the program, where original 40-min process is compressed within $\sim$ 30s. Consequently, parallel acceleration with 20 threads achieves $\times 80 (\pm 2)$ speedup to the benchmark, and processor efficiency is derived roughly 64% accordingly.

**Table 1:** Comparison between Evolution-focused MAS and case-focused MAS on single-thread mode

	Evolution-focused MAS	Case-focused MAS
Total Infected Agents	84%	84%
Cache References	$2.5 \times 10^{11}$	$1.5 \times 10^{10}$
Cache Misses	$8.5 \times 10^{10}$	$8 \times 10^{9}$
Running Time (s)	$2.6 (\pm 0.7) \times 10^3$	$6 (\pm 0.04) \times 10^2$
Speedup	$\times 1$	$\times 4.3 (\pm 0.1)$

## Conclusions

The MAS is an essential method for studying epidemiology sciences, especially for intervention for COVID-19 transmission. Traditional methods need querying over population as time evolves and suffers from poor-locality issues. In this work, we focus on optimizing MAS for infectious diseases, and propose two successive processes to accelerate computation. First, we reconstruct the iteration order of MAS and propose the case-focused method. It can improve data locality significantly, where 90% cache references are saved. Consequently, the program is accelerated by $\times 4.3$. Next, we design a thread-safe and high-performance hash table HHT for managing intermediate products from simulation. HHT leverages cache characteristics and SIMD instructions to optimize hash operations. It can further get a faster speed by 47% compared with classical hash table, and a 20-thread implementation achieves $\times 80$ speedup finally.

It should be noticed that major MAS researches adopted evolution-focused method as simulation backbone [60–62]. As indicated by [36], general MAS methods for infectious diseases have to handle irregular and randomly accessing agent states, which is a major factor limiting the calculation efficiency. Our proposed case-focused method overcomes their disadvantages to improve data locality significantly, and this method is suitable not only for COVID-19 but also for other infectious diseases. However, it takes a trade-off with blurred and delayed results. Although the results can be recovered by deconvoluting from a mixing kernel, kernel parameters need pre-calibrated under a specific scenario. Careful studies for this mechanism will be implemented in next works. Moreover, NPI and PI methods are crucial for intervening spreading of infectious diseases. Studies of fast calculation with these interventions are left in the future.

## Data Availability

Mobile phone data were provided by the Shenzhen Transportation Operation Command Center (Contact: Binliang Li, 240854198@qq.com). Travel survey data, building survey data and census data were offered by the Planning and Natural Resources Bureau of Shenzhen Municipality (Contact: Renrong Jiang, jiangrenrong@126.com). The epidemic surveillance data were provided by the Shenzhen Center for Disease Control and Prevention (Contact: Shujiang Mei, sjmei66@163.com). Researchers who meet the criteria for accessing to confidential data can send requests to the above local government departments. The daily confirmed cases of COVID-19 are publicly accessible from the Shenzhen Municipal Health Commission (http://wjw.sz.gov.cn/yqxx/). Baidu migration data can be openly obtained from http://qianxi.baidu.com/.

## Abbreviations

MAS: Multi-agent simulation
COVID-19: Coronavirus disease 2019
HHT: Hierarchical hash table
CHT: Cacheline hash table
SHT: Software hash table
BIT: Bulk-info table
CPU: Central processing unit
GPU: Graphics processing unit
FPGA: Field programmable gate array
SIMD: single instruction, multiple data
CDC: Center for disease control
SLIR: susceptible-latent-infectious-removed
